# On Dynamic Pitch Benefit for Speech Recognition in Speech Masker

**DOI:** 10.3389/fpsyg.2018.01967

**Published:** 2018-10-22

**Authors:** Jing Shen, Pamela E. Souza

**Affiliations:** ^1^Department of Communication Sciences and Disorders, Northwestern University, Evanston, IL, United States; ^2^Knowles Hearing Center, Northwestern University, Evanston, IL, United States

**Keywords:** speech recognition in noise, pitch perception, aging, hearing loss, cognition

## Abstract

Previous work demonstrated that dynamic pitch (i.e., pitch variation in speech) aids speech recognition in various types of noises. While this finding suggests dynamic pitch enhancement in target speech can benefit speech recognition in noise, it is of importance to know what noise characteristics affect dynamic pitch benefit, and who will benefit from enhanced dynamic pitch cues. Following our recent finding that temporal modulation in noise influences dynamic pitch benefit, we examined the effect of speech masker characteristics on dynamic pitch benefit. Specifically, the first goal of the study was to test the hypothesis that dynamic pitch benefit varies depending on the availability of pitch cues in the masker and the intelligibility of masker. The second goal of this study was to investigate whether older listeners as a group can benefit from dynamic pitch for speech recognition in speech maskers. In addition, individual factors of hearing loss and working memory capacity were examined for their impact on older listeners' dynamic pitch benefit. Twenty-three younger listeners with normal hearing and 37 older listeners with varying levels of hearing sensitivity participated the study, in which speech reception thresholds were measured with sentences in speech maskers. While we did not find an effect of masker characteristics on dynamic pitch benefit, the results showed older listeners can benefit from dynamic pitch for recognizing speech in speech maskers. The data also suggest that among those older listeners with hearing loss, dynamic pitch benefit is stronger for individuals with higher working memory capacity. This can be attributed to their ability to exploit facilitated lexical access in processing of degraded speech signal.

## Introduction

Pitch, as defined by the percept of fundamental frequency, is one of the most powerful acoustic cues in auditory perception. In speech, pitch is produced by the vibration of the vocal folds and varies naturally across time. This dynamic aspect of pitch carries prosodic information that facilitates speech recognition (Cutler, 1976; Steinhauer et al., 1999; Brown et al., 2011) and conveys emotion in speech (Fairbanks, 1940; Frick, 1985). Previous research has demonstrated that dynamic pitch cues aid speech perception in background noise (Laures and Bunton, 2003; Binns and Culling, 2007; Miller et al., 2010; Shen and Souza, 2017a). The data from these studies has consistently showed speech with natural dynamic pitch cues is better perceived than monotonous speech, when embedded in background noise. Reduction or inversion of pitch contour in target speech is detrimental for speech perception in noise, while strengthened dynamic pitch (by exaggerating the pitch contour) has not been found more beneficial when compared to preserving natural pitch contours.

While these effects have been demonstrated in a variety of noises, there is a tendency for dynamic pitch to be more helpful in speech maskers than non-speech noises (Laures and Bunton, 2003; Binns and Culling, 2007). This advantage from the speech maskers can at least be partially attributed to the temporal modulation of noise (Shen and Souza, 2017a). Following these findings, it was of interest to determine whether any additional features of the speech masker (i.e., intelligibility and availability of pitch cues) affects dynamic pitch benefit. In other words, once the factor of temporal modulation is controlled for, does a real speech masker provide more opportunity and/or pose more needs for dynamic pitch benefit than a non-speech masker?

### Dynamic pitch benefit for speech perception in speech maskers

The literature on speech-on-speech masking often uses the term “informational masking” to describe various masking phenomena that cannot be simply attributed to the overlap in spectral energy (e.g., Schneider et al., 2007; Shinn-Cunningham, 2008; Lutfi et al., 2013). It has been suggested that there are at least two sources of informational masking (e.g., Shinn-Cunningham, 2008; Rosen et al., 2013). One source taxes the auditory system by requiring the separation of multiple speech streams while the other interferes with listeners' attentive processing of the independent speech streams.

We know pitch information in speech facilitates stream segregation. Pitch has been shown to aid speech-on-speech perception by decreasing the similarity between the target and masker. As a result, listeners recognize continuous speech better in vocoded speech maskers when compared to real speech maskers (Vestergaard and Patterson, 2009; Ezzatian et al., 2011; Rosen et al., 2013). Intuitively, if dynamic pitch benefit stems from its role in facilitating stream segregation, any changes in the difficulty of stream segregation task will potentially affect dynamic pitch benefit. This rationale is also motivated by previous data showing that dynamic pitch is beneficial in speech-on-speech scenarios, particularly when the pitch of the speech masker is close to the pitch of the target, posing a difficult stream segregation scenario (Assmann, 1999). In this study, Assmann used sentence pairs that were produced by the same male talker and manipulated the pitch variation of both target and masker sentences. An 8% increase in speech intelligibility was found when sentences of the target and masker had pitch variation, and when the average pitch of the target and masker sentences was fairly close (0 or 1 semitones apart). Because this study prioritized a tight control over other acoustic cues by using the same talker for target and masker speech, such a setup provides limited information as to what the role of dynamic pitch is when the masker and target speech have different perceptual characteristics, as would be the case in a realistic listening scenario. Using a more realistic stimuli setup, the present study examines whether degraded pitch cues of the masker, which decreases perceptual similarity to the target, affects dynamic pitch benefit for the recognition of target speech.

With regard to the higher-level processing mechanisms, we know dynamic pitch cues can facilitate processing of linguistic information in target speech by providing prosodic cues (e.g., Cutler, 1976; Brown et al., 2011). If dynamic pitch benefit stems from a release of higher-level processing resource, this facilitation effect can be stronger in intelligible speech maskers as compared to in non-speech noises. To this end, a study by Binns and Culling (2007) tested dynamic pitch benefit, using different male talkers for target and masker speech stimuli and thereby creating a real speech masker scenario. Their data indicated that younger listeners benefit more from dynamic pitch with an intelligible single-talker speech masker, as compared to unintelligible speech-shaped noise. This result, however, may be attributed to temporal modulation in the speech masker, to masker intelligibility, or to both factors. While temporal modulation in a speech masker is known to modulate dynamic pitch benefit (Shen and Souza, 2017a), the question that has yet to be examined is whether intelligibility of the speech masker influences dynamic pitch benefit when the temporal characteristic is controlled for.

The present study tested these two questions by using two processed speech masker conditions. When compared against the unaltered speech masker, each of the processed masker conditions reserved one of the two features of the unaltered speech, pitch cues and intelligibility. One masker condition was a 32-channel vocoded speech masker with degraded pitch cues but was highly intelligible, while the other was a time-reversed speech masker with pitch cues preserved that was unintelligible.

### Older individuals' dynamic pitch benefit for speech recognition in speech maskers

Our recent data has demonstrated that older listeners can benefit from dynamic pitch cues in non-speech noise (Shen and Souza, 2017a). This evidence suggests that older listeners as a group are able to utilize dynamic pitch in speech perception, particularly when the noise has temporal modulation. The present study continues this line of inquiry, with a focus on the scenario of speech maskers. Specifically, we ask the question: to what extent can older listeners benefit from dynamic pitch cues and recognize target speech in speech maskers?

We know the majority of older listeners have difficulty recognizing speech when the masker is also speech (e.g., Divenyi and Brandmeyer, 2003; Gordon-Salant and Fitzgibbons, 2004; Rajan and Cainer, 2008). This problem has been attributed to older listeners' poor ability to utilize acoustic cues for perceptually segregating the speech streams (Murphy et al., 2006; Helfer and Freyman, 2008). For instance, Helfer and Freyman found that older listeners with a variety of hearing abilities performed more poorly than younger listeners in a speech-on-speech task. After adjusting for baseline performance, the older group had specific difficulty when the target and masker consisted of speech from talkers of different genders, which differ in terms of acoustic characteristics (i.e., overall pitch, voice quality). Drawing upon this rationale, one of the objectives of the present study was to examine the effect of masker characteristics on older listeners' dynamic pitch benefit. If older listeners are less capable of exploiting the different characteristics in maskers (including degraded pitch cues or unintelligible masker) for stream segregation, dynamic pitch cues may be reduced in the processed masker conditions.

While earlier data from younger listeners with normal hearing did not support the benefit from strengthening dynamic pitch cues in target speech by exaggerating pitch contours (Miller et al., 2010; Shen and Souza, 2017a), data from older listeners with significant hearing loss demonstrated substantial variability across individuals in terms of which dynamic pitch strength was optimal for speech recognition (Shen and Souza, 2017b). Following previous findings, the current study included a strengthened dynamic pitch condition, as well as additional analyses focusing on this subgroup of older listeners.

### Individual factors that influence older listeners' dynamic pitch benefit

Considering the variability across older individuals in terms of dynamic pitch benefit (as demonstrated by the previous studies), it is important to know who is likely to benefit and what the predictors for dynamic pitch benefit in the speech masker are if dynamic pitch were to be enhanced to facilitate speech recognition in noise. It is one of our goals to examine how older individuals' dynamic pitch benefit is influenced by two factors that are known to have an impact on speech-in-noise ability, namely hearing sensitivity and working memory capacity.

Older individuals' speech-in-noise difficulty stems from multiple factors, such as age-related changes in hearing and cognitive abilities (Humes, 1996; Frisina and Frisina, 1997; Pichora-Fuller and Souza, 2003). First, the amount of hearing loss an individual has influences their difficulty recognizing speech in noise (Helfer and Wilber, 1990; Stuart and Phillips, 1996). For listeners with hearing loss, hearing sensitivity has been found to strongly predict the ability to recognize speech in noise, even after controlling for audibility effects (e.g., George et al., 2007). Our recent data suggest that, for older individuals, hearing loss (indicated by audiometric thresholds) explains a significant proportion of the inter-subject variability in dynamic pitch benefit. More hearing loss is associated with less dynamic pitch benefit in non-speech noise (Shen and Souza, 2017a). In the present study, we extend this inquiry by asking whether the amount of hearing loss influences dynamic pitch in speech maskers that have various perceptual characteristics.

In addition to hearing ability, older individuals' cognitive abilities (e.g., working memory capacity, executive function, attention) have also been shown to modulate speech in noise performance (Humes et al., 2006; Sörqvist and Ronnberg, 2012; Füllgrabe et al., 2015; McLaughlin et al., 2018). Specifically, working memory capacity, which is defined as the ability to process and store information simultaneously (Baddeley, 1992), is known to be an influential factor that mediates speech perception in noise and under other adverse conditions (Akeroyd, 2008; Besser et al., 2013). Specifically, individuals with higher working memory capacity understand speech better in noisy environments, which can be explained by the Ease of Language Understanding (ELU) model (Rönnberg et al., 2013). According to the ELU model, the signal degradation induced by background noise creates a mismatch between the phonological presentations from the speech input and the listener's long-term memory. Under this circumstance, more explicit cognitive processing is required and working memory resources are heavily taxed. Individuals who have higher working memory capacity therefore have more resources available for this explicit processing and can recover the speech information better than those with lower working memory capacity.

When a speech-on-speech masking scenario is considered, we know younger listeners with high working memory capacity understand and remember target speech more accurately when it is imbedded within a speech masker compared to non-speech masker (Sörqvist and Ronnberg, 2012). This finding indicates that speech-on-speech masking heavily taxes working memory capacity by engaging its key components of central executive control (Baddeley, 2000) and inhibition to the masker interference (Miyake et al., 2000). Taken with the findings showing the impact of aging on working memory capacity (e.g., Hasher and Zacks, 1988), it is not surprising that working memory capacity is strongly associated with speech perception in speech maskers for older listeners (see Besser et al., 2013 for a review). Presumably, the higher working memory capacity an individual has, the better he/she can exploit acoustic cues (including dynamic pitch) to separate speech streams and ignore the interference from a masker. Following from these findings, it is anticipated that the measure of working memory capacity is a strong predictor for the influence of the dynamic pitch cue in the speech masker.

The second objective of this study was therefore to examine the following questions in a group of older listeners with a variety of hearing levels. First, can older listeners, particularly those with significant hearing loss, benefit from dynamic pitch for speech recognition in speech maskers? Second, do masker characteristics (i.e., presence of pitch cues and intelligibility) affect older listeners' dynamic pitch benefit? Last, do individual factors (i.e., hearing sensitivity and working memory capacity) modulate older listeners' dynamic pitch benefit?

## Methods

### Participants

For the younger group, 23 younger adults (16 female and 7 male) ages 18–31 years (mean age 22.3 years) participated in this study. All the listeners had normal hearing (pure tone threshold ≤ 20 dB hearing level (HL) at all octave frequencies between 250 and 8,000 Hz.

The older group consisted of 37 older adults (20 female and 17 male) aged 57–84 years (mean age 71.8 years). They had various levels of hearing ranging from near normal hearing to mild-moderate hearing loss. Based on their pure tone threshold (PTA, average of thresholds at.5, 1, 2 kHz), the older listeners were further divided into two groups: 18 older listeners with near-normal hearing (all the listeners have PTA ≤ 20 dB HL, with PTA group mean of 12.5 dB HL), and 19 older listeners with hearing loss (all the listeners have PTA > 20 dB HL, with PTA group mean of 33.42 dB HL). Among the 19 older listeners with hearing loss, 5 of them were hearing aid users.

The participants were recruited via newspaper advertisement and flyers in the Greater Chicago area. All participants were native English speakers. None of the participants had experience in tonal languages, or more than 3 years of musical training. We intentionally included these criteria regarding language and music experience in order to control for any potential variability in processing of fundamental frequency and speech perception in noise (e.g., Coffey et al., 2016; Presacco et al., 2016), which stems from experience-shaped difference in neural encoding mechanism (e.g., Wong et al., 2007; Bidelman et al., 2009).

Older participants were screened for mild cognitive impairment by using the Montreal Cognitive Assessment (Nasreddine et al., 2005). All participants passed the test with a cutoff score of 23, which has been shown to maximize the test's sensitivity and specificity (Lee et al., 2008; Luis et al., 2009). The older group also had an additional measure of working memory capacity using the Reading Span Test (Daneman and Carpenter, 1980; Rönnberg et al., 1989). This test was designed to measure individual working memory capacity in terms of coordinating storage and processing needs simultaneously. During the test, 54 sentences were shown on the computer screen one word or word pair at a time, with on-screen duration of 800 msec. Half of the sentences were absurd and half were semantically meaningful. The participants were asked to read each sentence and make a semantic judgment about whether the sentence makes sense. After each block of sentences (3–6 sentences per block), the participants were asked to recall the first or the last words of the last presented block of sentences. The measure of the individual's working memory capacity was the percentage of words that were correctly recalled.

The study consisted of a single 2-h session and participants were paid for their time. The study protocol was approved by the Institutional Review Board at Northwestern University.

### Stimuli

The target speech stimuli were drawn from PRESTO sentences (Gilbert et al., 2013) and produced by a male talker. Each sentence had between 3–6 key words. These sentences have low predictability and therefore were ideal for minimizing the use of linguistic context for speech recognition. The dynamic pitch contours of the stimuli were manipulated and the sentences were resynthesized using the PRAAT program (Boersma and Weenink, 2013) with the method of Pitch-Synchronous Overlap-and-Add (PSOLA, Moulines and Charpentier, 1990). The purpose of this manipulation was to change the dynamic pitch (fundamental frequency, f0) contour of the sentence while keeping other prosodic cues constant (e.g., duration, intensity). Three f0 conditions were created for each sentence by using the following formula.

(1) $$\begin{array}{l} \text{Instant f}0=\text{Sentence average f}0+(\text{Original instant f}0\\ \;-\text{Sentence average f}0)\times \text{Pitch factor.} \end{array}$$

The pitch factor was set to 0 in the monotone condition, 1 in the original pitch condition, and 1.75 in the strong pitch condition (Miller et al., 2010; Shen and Souza, 2017a).

A two-talker babble was used as masker speech due to its reduced variability compared to a one-talker masker (Rosen et al., 2013). The speech material was drawn from a recoded passage of “North Wind and Sun,” with different segments from a female talker and a male talker. Speech materials were edited to remove pauses longer than 250 msec. The signals were normalized to the same root-mean-square (RMS) before being digitally added together.

The three masker conditions were the unaltered condition, the 32-channel vocoded condition, and the time-reversed condition. The original babble was used for the unaltered condition. The 32-channel noise-vocoded babble was created following the vocoding method reported in Rosen et al. (2013). The original babble signal was digitally reversed in time to create the time-reversed condition. All three masker signals were RMS normalized before being used in the experiment.

To create 32-channel noise-vocoded babble, the original babble was processed using a customized MATLAB program (Mathwork, Natick, MA). The babble was digitally filtered into 32 bands, using fourth-order Butterworth infinite impulse response filters. Filter spacing was based on equal basilar membrane distance (Greenwood, 1990) across a frequency range of 0.1–5 kHz. In order to extract the amplitude envelope, the output signal from each band was full-wave rectified and low-pass filtered at 30 Hz. The cutoff frequency was set to be 30 Hz to exclude quasi-periodic signals that may provide pitch cues (Rosen, 1992). The envelope was then multiplied by a wide-band noise carrier, and the resulting signal was filtered using the same 32-band band-pass filter that was used in the first stage of filtering. The RMS level was adjusted at the output of the filter to match the original level in that band, before the signals were summed up across bands.

### Procedure

Prior to experimental testing, participants completed an audiometric battery consisting of case history, otoscopy, pure tone threshold testing, and word recognition in quiet with NU-6 25-word lists (Tillman and Carhart, 1966). The audiometric testing was done using an AC40 Interacoustics audiometer connected to ER-3 insert earphones.

Using a customized MATLAB program (Mathwork, Natick, MA), speech reception thresholds (SRTs) were obtained for all nine conditions (3 target pitch conditions × 3 masker conditions) with an adaptive procedure (Plomp and Mimpen, 1979) in which masker level varied aross trial. The initial SNR increment was 6 dB until at least half of the keywords were repeated correctly. For each subsequent sentence, the SNR increased by 2 dB when fewer than half of the key words were correctly repeated or decreased by the same amount for more than half of the correct key words. The number of trials was fixed at 15, tracking 50% correct. SRTs for each condition were measured twice. A different set of sentences was used to retest a condition when fewer than 3 reversals were obtained, or when the standard deviation across the final reversals exceeded 4 dB. Thresholds for each run were computed by taking the mean SNR (dB) across the reversals at the final step size of 2 dB. Participants were given brief practice on the different conditions to familiarize them with the different types of speech and noise. Practice consisted of 9 trials and started at 6 dB SNR. The order of conditions in the experiment was counterbalanced across participants following a Latin square design. Stimuli (i.e., target embedded in masker) were presented monaurally in the better ear at 68 dB SPL. The better ear was defined as the ear with lower pure tone threshold (PTA, i.e., better hearing). In the case that two ears had same PTA, one ear was randomly picked for testing.

To accommodate each individual's hearing threshold, stimuli were amplified using the National Acoustics Laboratories-Revised (NAL-R) linear prescriptive formula based on individual thresholds (Byrne et al., 2001). Stimuli were presented using an M-Audio FastTrackPro external soundcard (M-Audio) and an ER-2 insert earphone (Etymotic Research, Elk Grove, IL) in the test ear. Hearing aid users were tested without their hearing aids. Listeners were seated in a double-wall sound-treated booth. They were instructed to repeat the sentences aloud for the experimenter to score.

While the 32-channel vocoded speech is highly intelligible, the effect of exposure may increase the intelligibility of the vocoded masker over the period of the speech recognition testing (e.g., Davis et al., 2005), which could affect the perception of target speech. To control for this possible confounding factor, an intelligibility verification process was included in the protocol. Spoken sentences from IEEE corpus (Rothauser et al., 1969) were processed using the same vocoding method as for the masker. Speech recognition performance without any background noise was assessed twice using 10 vocoded sentences each time, both before and after the main study protocol for measuring SRTs. The percent correct scores (younger group 95.58% before testing, 94.08% after testing; older group 85.24% before testing, and 84.86% after testing) were compared using *t*-tests and were not significantly different before and after testing [*p* > 0.1 for all the comparisons, younger: *t*_(22)_ = 1.26, older: *t*_(36)_ = 0.28].

## Results

Data analysis was conducted using mixed-effects linear regression implemented with R (R Core Team-Version 3.2.1). Mixed effects linear regressions were conducted using R's lme4 and lmerTest libraries (Kuznetsova et al., 2013; Bates et al., 2014).

### Effect of masker and pitch conditions on speech recognition

**Figure 1:**
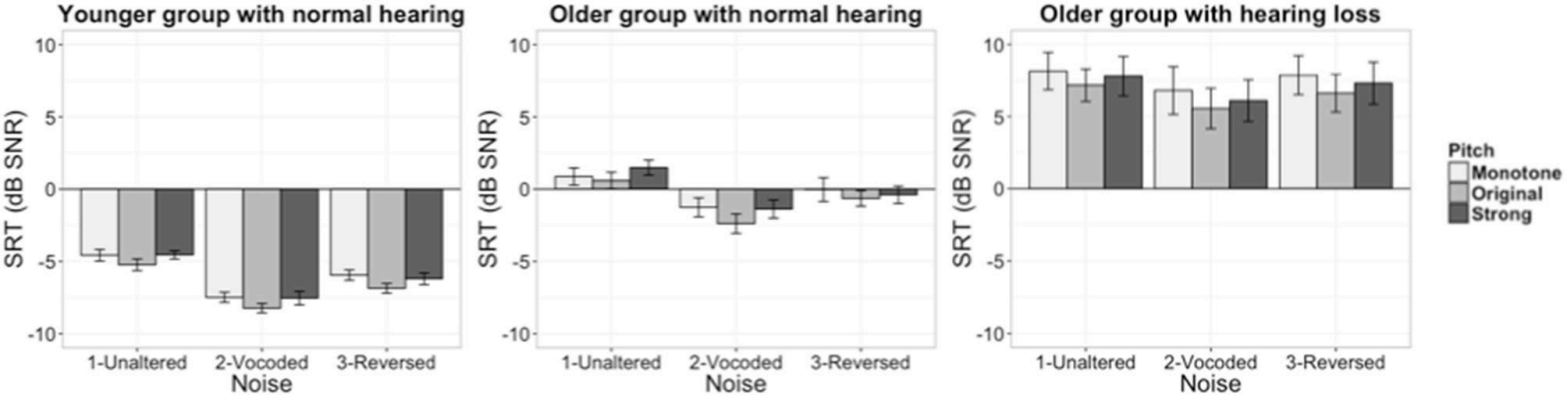
Speech Reception Threshold scores of all listener groups (left panel: younger group with normal hearing, middle panel: older group with near-normal hearing, right panel: older group with hearing loss. Error bars present ± 1 standard error).

The SRT values of the three groups (younger, older normal hearing, and older hearing loss) are presented in Figure 1. The first model was built to examine three questions: (1) Do listeners recognize speech better in Original/Strong pitch conditions as compared to Monotone condition? (2) Do listeners recognize speech better when speech masker is pitch-degraded, or is unintelligible? (3) Does overall speech-in-noise performance vary depending on groups (younger, older normal hearing, and older hearing loss)? The dependent variable was speech recognition in noise performance (as indicated by SRT). The model included fixed effects of pitch strength condition (monotone, original, strong), masker condition (unaltered, vocoded, time-reversed), group (younger, older normal hearing, older hearing loss), and test block order (to control for order effects), as well as random by-participant intercept. As all the fixed effects (pitch, masker, group) are categorical variables, simple coding strategy was used to compare each level of a variable to a reference level. Specifically, the target dynamic pitch condition was coded to compare SRTs between the Monotone condition and the Original pitch condition; and also to compare SRTs between the Monotone condition and the Strong pitch condition. Masker condition was coded to compare SRTs between the Unaltered masker and the Vocoded masker conditions; and also to compare SRTs between the Unaltered masker and the Time reversed masker. Group was simple coded to first compare older near-normal hearing group with younger group, and also to compare older hearing loss group with older near-normal hearing group. Block order was also simple coded to control for practice effect across two test runs.

Table 1 provides a summary of the significance and *p*-values based on the χ^2^ test of the change in deviance between the models with and without the predictor of interest. Both the masker and pitch conditions (but not the interaction) significantly improved the model. Group as a fixed factor also significantly improved the model. All the predictors (in terms of detailed contrasts, same for all the effects hereafter) in the final model were tested using Wald test. Results suggest that perception of the target speech was facilitated in both processed masker conditions, as compared to the unaltered masker: Vocoded masker (b = −2.42, SE = 0.15, *t* = −16.44, *p* < 0.001); Time reversed masker (b = −1.13, SE = 0.15, *t* = −7.70, *p* < 0.001). Speech recognition performance was significantly better in the Original pitch condition as compare to Monotone condition (b = −0.86, SE = 0.15, *t* = −5.86, *p* < 0.001) but did not differ between Strong pitch condition and Monotone condition (b = −0.19, SE = 0.15, *t* = −1.31, *p* > 0.1). In terms of group difference on speech recognition performance, younger listeners did significantly better (lower SRTs) than older listeners with near-normal hearing (b = −5.94, SE = 1.13, *t* = −5.22, *p* < 0.001), who also significantly outperformed older hearing loss group (b = 7.38, SE = 1.18, *t* = 6.21, *p* < 0.001). The effect of block order was also significant in the direction that listeners did better with first block of each condition better than the second one (b = −0.41, SE = 0.12, *t* = −3.41, *p* < 0.001), which could be explained by the potential fatigue effect in completing the speech-in-noise task.

**Table 1:** Model comparison χ^2^ and *p*-values for speech recognition in maskers (*n* = 60).

	**All three groups SRT**		
**Variable**	**Model comparison χ^2^**	**Degree of freedom**	**Model comparison *p*-value**
Dynamic pitch	37.29	2	<0.001
Masker condition	241.16	2	<0.001
Dynamic pitch × masker condition	2.27	4	>0.1
Group	74.91	2	<0.001
Block order	11.67	1	<0.001

The data are consistent with the literature in demonstrating the effect of reduced masking in the 32-channel vocoded and time-reversed speech maskers, as compared to the unaltered masker. This finding supports the view that informational masking stems from two sources of interference (Shinn-Cunningham, 2008; Rosen et al., 2013). Presumably, the target and masker speech sound less similar when the periodicity cues are degraded by vocoding and listeners are able to utilize this difference to recognize target speech better (Ezzatian et al., 2011). Similarly, the time-reversed speech masker had reduced masking as compared to intelligible speech masker in the unaltered condition. This also aligns with the speech perception literature, showing the effect of masker intelligibility (e.g., Festen and Plomp, 1990; Summers and Molis, 2004).

### Effect of masker and pitch conditions on dynamic pitch benefit

**Figure 2:**
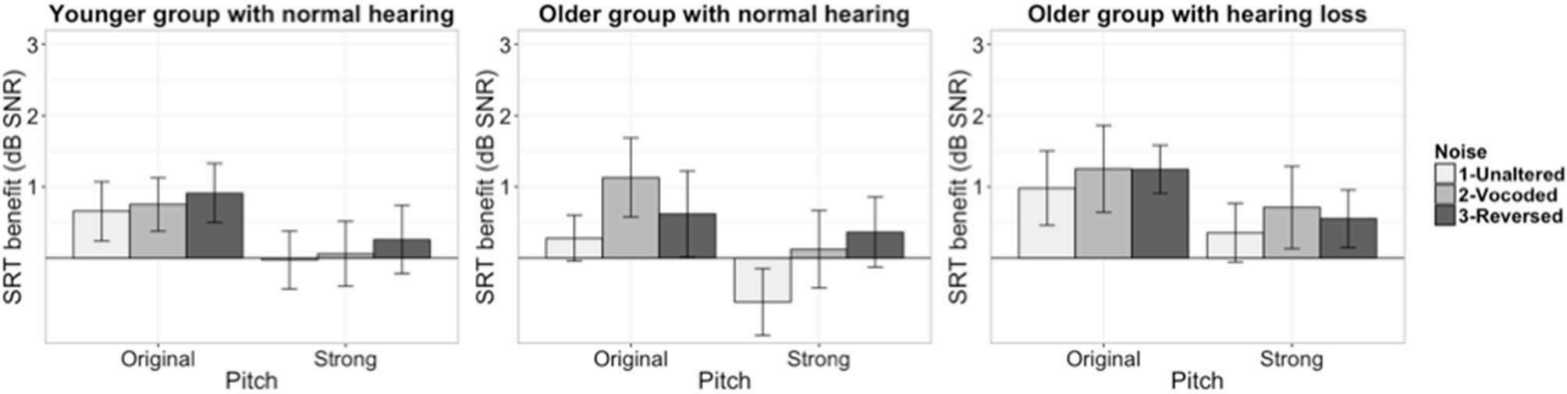
Dynamic benefit scores of all listener groups (left panel: younger group with normal hearing, middle panel: older group with near-normal hearing, right panel: older group with hearing loss. Error bars present ± 1 standard error).

The second model was built to evaluate the effect of noise manipulation on dynamic pitch benefit (i.e., the difference between the SRTs of the Monotone condition and the Original/Strong pitch conditions). The dependent variable was dynamic pitch benefit. Figure 2 illustrates the SRT benefit scores for younger and older groups. The model included fixed effects for masker condition, pitch condition, group, and random by-participant intercepts. The SRT score in the original pitch/unaltered masker condition, which could potentially influence the amount of benefit the listeners get from dynamic pitch cues (Bernstein and Grant, 2009), was also included in the model as a baseline measure. Dynamic pitch condition was coded using simple coding strategy to compare between Original and Strong dynamic pitch conditions. Masker condition was also simple coded following the same strategy used in the speech recognition analysis. The group was coded with two comparisons: younger normal hearing group vs. older near-normal hearing group, older near-normal hearing group vs. older hearing loss group.

Model comparison results are reported in Table 2. Only the dynamic pitch condition but neither the masker condition, nor the interaction significantly improved the model. The summary of the final model showed that the amount of benefit was larger for the Original compared to Strong dynamic pitch conditions (b = −0.67, SE = 0.21, *t* = −3.14, *p* < 0.01). The listeners' dynamic pitch benefit scores were not significantly different between the unaltered masker and the vocoded/time reversed masker conditions (p>0.1). In terms of the overall amount of dynamic pitch benefit, neither the younger group nor the older hearing loss group was significantly different from the older near normal hearing group (*p* > 0.1).

**Table 2:** Model comparison χ^2^and *p*-values for dynamic pitch benefit across all three groups (*n* = 60).

	**All groups dynamic pitch benefit score**		
**Variable**	**Model comparison χ^2^**	**Degree of freedom**	**Model comparison *p*-value**
Dynamic pitch	9.82	1	<0.01
Masker condition	2.74	2	>0.1
Group	1.59	2	>0.1
Dynamic pitch × masker condition	0.17	2	>0.1
Baseline speech recognition	1.51	1	>0.1

As older listeners usually have more difficulty recognizing speech in background noise than younger listeners, it is of particular interest whether the two groups of older individuals can benefit from dynamic pitch cues. When dynamic pitch benefit was examined in each of the two older groups (near-normal hearing and hearing loss) using the same model structure, with target pitch condition, masker condition, and baseline SRT as fixed factors, and random by-participant intercepts. The results showed that neither target pitch condition nor masker condition significantly improved model fit in predicting the amount of dynamic pitch benefit (see Table 3 for a summary of the significance and *p*-values based on the χ^2^ test of the change in deviance between the models with and without the predictor of interest). These results suggest that, older listeners in general do not benefit more from original dynamic pitch as compared to stronger dynamic pitch.

**Table 3:** Model comparison χ^2^ and *p*-values dynamic pitch benefit of the older near-normal hearing group (*n* = 18) and the older group with hearing loss (*n* = 19).

	**Older near-normal hearing group**			**Older hearing loss group**		
**Variable**	**Model comparison χ^2^**	**Degree of freedom**	**Model comparison *p*-value**	**Model comparison χ^2^**	**Degree of freedom**	**Model comparison *p*-value**
Dynamic pitch	3.29	1	>0.05	2.69	1	>0.1
Masker condition	3.08	2	>0.1	0.50	2	>0.1
Dynamic pitch × masker condition	0.71	2	>0.1	0.02	2	>0.1
Baseline speech recognition	5.07	1	< 0.05	0.80	1	>0.1

### Influence of individual factors on dynamic pitch benefit

One of the goals of this study was to examine the impact of hearing (i.e., PTA), and working memory capacity on older listeners' dynamic pitch benefit. We examined this question in the two subgroups of older listeners, respectively, one with near normal hearing and one with hearing loss. Due to the fact that none of the fixed factors were significant in the previous analysis (*p* > 0.05 for all conditions), simple linear regression was used for this analysis. In both groups, PTA and working memory capacity were not correlated [older normal hearing: *r* = −0.04, *t*_(16)_ = 0.18, *p* > 0.1; older hearing loss: *r* = 0.1, *t*_(15)_ = 0.40, *p* > 0.1]. Therefore, the models were built with PTA and working memory capacity as independent variables, with the dependent variable being dynamic pitch benefit under multiple noise conditions (baseline performance of speech recognition in speech masker was also controlled for in the model).

In the group of older individuals with near-normal hearing, neither PTA nor working memory capacity was found to be a significant predictor for mean dynamic pitch benefit (PTA: b = −0.007, SE = 0.02, *t* = −0.309, *p* > 0.1; Working memory capacity: b = 0.026, SE = 0.016, *t* = 1.615, *p* > 0.1). When the impact from PTA and working memory capacity was assessed in each of the three masker conditions, neither factors significantly predicted dynamic pitch benefit in any of the three masker conditions (*p* > 0.1 for all masker conditions),

The same set of analyses was used to examine the data from the group of older listeners with hearing loss. Neither of the individual factors significantly predicted the mean dynamic pitch benefit score across three masker conditions (PTA: b = −0.005, SE = 0.014, *t* = −0.404, *p* > 0.1; Working memory capacity: b = −0.009, SE = 0.013, *t* = −0.726, *p* > 0.1). For each of the masker conditions, working memory capacity was found a significant predictor for dynamic pitch benefit in the unaltered speech masker (b = 0.14, SE = 0.05, *t* = 2.62, *p* < 0.05) and in the vocoded speech masker (b = 0.18, SE = 0.08, *t* = 2.11, *p* = 0.05). Neither of the individual factors significantly predicted the dynamic pitch benefit in the time-reversed masker conditions (*p* > 0.1).

## Discussion

### Characteristics of speech masker and dynamic pitch benefit

One of the main objectives of this study was to examine the effect of speech masker on dynamic pitch benefit in both younger and older listeners. It was hypothesized that two sources of interference in speech masker can influence dynamic pitch benefit, namely availability of pitch cues and intelligibility. Data from younger listeners with normal hearing and older listeners with a wide range of hearing ability do not support this hypothesis and show that neither of these two factors affects dynamic pitch benefit for speech recognition in these listeners. It is worth noting that these results occurred in the presence of the release from speech masking in the two processed maskers (32-channel vocoded and time-reversed). This finding suggests that dynamic pitch benefit for speech recognition in speech masker is not contingent on the demand of the stream segregation task, which can be altered by changes in the masker characteristics. In other words, while the signal processing reduces the amount of informational masking from the speech masker, it does not affect the opportunity and/or needs to exploit dynamic pitch cues in target speech. Taken together with the previous finding that there is increased dynamic pitch benefit when noise has stronger temporal modulation (Binns and Culling, 2007; Shen and Souza, 2017a), the evidence so far has suggests that dynamic pitch benefit is primarily driven by the availability of the dynamic pitch cues against background noise, but not affected by the characteristics of the masker that were tested in this study.

Specifically, as demonstrated by Binns and Culling (2007), dynamic pitch benefit (i.e., SRT difference between monotonous speech and natural dynamic pitch condition) was about 1.6 dB higher in 1-talker speech masker than in steady-state noise. Our data from younger listeners with normal hearing showed that, on average, there was a 1.8 dB increase in dynamic pitch benefit in non-speech noise with 1-talker temporal modulation, as compared to steady-state (1-talker speech-shaped) noise (Shen and Souza, 2017a). Given the alignment between these results, it appears that the difference observed in Binns and Culling is likely due to the effect from temporal modulation difference between the two noises (i.e., 1-talker speech masker vs. speech-shaped noise). Our present data provide support for this interpretation by suggesting that when temporal modulation is controlled for, characteristics of the masker (i.e., intelligibility and pitch cues) do not significantly influence dynamic pitch benefit.

### Older listeners' dynamic pitch benefit in speech maskers

One of the questions asked in the present study was that whether older listeners (either with near-normal hearing or have hearing loss) can benefit from dynamic pith cues in speech maskers. To this end, our data demonstrated that both older groups were able to benefit from dynamic pitch and the magnitude of their benefit on a group level was comparable to that of the younger group with normal hearing. It is an interesting finding given that older listeners' speech recognition in noise performance was in general poorer than younger listeners, which is consistent with the literature (e.g., Helfer and Freyman, 2008). While there is no obvious explanation for this finding, a possible interpretation is that dynamic pitch benefit does not vary substantially once temporal modulation in noise is held constant (i.e., all the speech maskers had comparable amount of temporal modulation) and the listeners are able to utilize the temporal modulation in noise (likely for the younger and older normal hearing groups). While older listeners with hearing loss, as a group, was not different than their peers with near-normal hearing, cognitive ability may be compensating for the declined hearing ability on an individual level (which will be discussed later in this section).

### Effect of strengthened dynamic pitch cues

In regard to the effect of dynamic pitch strength on benefit, our data support previous results in showing the limited benefit from strengthened dynamic pitch (Miller et al., 2010; Shen and Souza, 2017a) in younger listeners with normal hearing and older listeners with a variety of hearing levels. The group of older listeners with significant hearing loss had comparable performance levels in original and strong dynamic pitch conditions, with substantial variability across individuals. Taken together with the previous findings, it appears the benefit from this type of dynamic pitch enhancement is limited for most listeners with normal or near-normal hearing. On the other hand, it should be noted that while this dynamic pitch enhancement is the only method that has been examined, the efficacy of enhancing pitch cues with other methods has yet to be examined. For instance, we know from the psycholinguistic literature that some dynamic pitch cues are linguistically more important than others for online processing of speech (e.g., Brown et al., 2011). Future work can shed light on this possibility by examining whether enhancement strategies that focus on those linguistically meaningful pitch cues could improve listeners' speech perception in background noise.

### Individual factors that influence older listeners' dynamic pitch benefit

Driven by the ultimate goal of improving older listeners' speech perception with dynamic pitch cues, we were interested in the listener factors that may influence individuals' dynamic pitch benefit. In this study, hearing threshold and working memory capacity were included to represent the impact of hearing and cognitive abilities. This probe yielded two noteworthy findings. First, in a group of older individuals who have significant hearing loss, those with higher working memory capacity benefit more from dynamic pitch for speech recognition in a real speech masker. Second, amount of hearing loss (as indicated by pure tone thresholds) was not a strong predictor for older individuals' dynamic pitch benefit in speech maskers.

First, our data suggest a significant impact of working memory capacity on dynamic pitch benefit, for older listeners with significant hearing loss. Those older individuals with higher working memory benefit more from dynamic pitch cues in the speech masker than those with lower working memory. To our knowledge, our data demonstrated, for the first time, the influence of cognitive ability on older listeners' dynamic pitch benefit for speech perception in noise. The significance of this finding is two-fold. First, it contributes to the current literature on the role of working memory capacity in speech perception under adverse conditions (Akeroyd, 2008; Rönnberg et al., 2010; Besser et al., 2013) by showing working memory also mediates the benefit from acoustic cues for speech perception under adverse conditions. For example, our results echo the previous finding that listeners with higher working memory capacity benefit more from temporal modulation in noise and glimpse target speech better (George et al., 2007). It is worth noting that dynamic pitch as a supra-segmental cue does not bear lexical information in English but may facilitate lexical access in continuous speech (e.g., Cutler, 1976). Therefore, according to the ELU model (Rönnberg et al., 2013), listeners with higher working memory capacity are able to utilize this cue during the explicit processing stage when speech signal is degraded by noise. Second, it is important to note that this relationship was observed only in the most adverse conditions of speech-on-speech masking (i.e., when masker was intelligible speech). This result indicates that dynamic pitch enhancement has the potential for improving speech perception in realistic noisy environment, for those older listeners who have significant hearing loss and high working memory capacity. Future research effort should be devoted to investigating this possibility.

Further, it is an interesting observation that the amount of hearing loss did not influence older listeners' dynamic pitch benefit in the present study. This finding appears to be inconsistent with our recent data that demonstrated a negative effect of hearing loss on dynamic pitch benefit in older listeners with significant hearing loss (Shen and Souza, 2017a). However, it is important to note two methodological choices in these two studies. First, the previous study used non-speech noises that had various degrees of temporal modulation, while the present study used 2-talker speech maskers that are mostly intelligible. With non-speech maskers, we found the critical factor that influences dynamic pitch benefit was whether a listener could perceive and utilize dynamic pitch cues across noise interruption, which was likely modulated by degree of hearing loss. In a speech-on-speech scenario, the speech recognition task taxes higher-level cognitive processing more heavily. Therefore, instead of hearing loss, cognitive ability (such as working memory capacity) becomes more critical for listeners to benefit from dynamic pitch to recognize target speech in speech masker, which was observed in the present dataset. Another piece of evidence that supports this explanation is that inter-subject variability becomes more homogenous across younger and older groups, as compared to our previous data (Shen and Souza, 2017a). The standard deviation in the older hearing loss group was 67% of the one in the younger group in the previous study but they become comparable in magnitude in the present study (2.08 and 2.10 dB SNR, respectively). When working memory capacity plays a stronger role in a speech-on-speech scenario, the variability in this cognitive ability is expected to manifest itself in the dynamic pitch benefit scores. We know working memory capacity varies across individuals in both younger and older adults (Souza and Arehart, 2015), which can potentially explain the comparable inter-subject variability in dynamic pitch benefit that we observed in the older and younger groups. Further, in both studies, hearing ability has been indicated by pure tone threshold. It is also possible that this measure does not capture those supra-threshold hearing abilities (such as perception of frequency modulation), which may have a more direct impact on dynamic pitch benefit in temporally modulated maskers. In future work, it will be important to examine the relationship between supra-threshold hearing measures and dynamic pitch benefit. From a clinical perspective, this line of work can also be extended to include a group of younger and mid-aged listeners who have hearing loss to examine the impact of hearing loss on dynamic pitch benefit for speech recognition in noise.

## Ethics statement

This study was carried out in accordance with the recommendations of Northwestern University research policy and guideline with written informed consent from all subjects. All subjects gave written informed consent in accordance with the Declaration if Helsinki. The protocol was approved by the Northwestern University Institutional Review Board.

## Author contributions

JS designed the study, collected the data, and wrote the manuscript. PS designed the study and wrote the manuscript.

### Conflict of interest statement

The authors declare that the research was conducted in the absence of any commercial or financial relationships that could be construed as a potential conflict of interest.
